# Activation of peroxymonosulfate with ZIF-67-derived Co/N-doped porous carbon nanocubes for the degradation of Congo red dye

**DOI:** 10.1038/s41598-024-62029-8

**Published:** 2024-05-29

**Authors:** Aya Khamis, Aya S. Mahmoud, Ahmed O. Abo El Naga, Seham A. Shaban, Nadia A. Youssef

**Affiliations:** 1 Chemistry Department, Faculty of Women, Ain Shams University, Cairo, Egypt; 2 Catalysis Department, Refining Division, Egyptian Petroleum Research Institute, Nasr City, 11727 Cairo Egypt

**Keywords:** Co nanoparticles, Metal–organic frameworks, Peroxymonosulfate activation, Congo red, Environmental sciences, Chemistry

## Abstract

In this study, porous carbon nanocubes encapsulated magnetic metallic Co nanoparticles (denoted as Co@N-PCNC) was prepared via pyrolyzing ZIF-67 nanocubes precursor at 600 °C and characterized by various technologies. It was used to activate peroxymonosulfate (PMS) to degrade Congo red (CR) dye efficiently. Over 98.45% of 50 mg L^−1^ CR was degraded using 0.033 mM PMS activated by 75 mg L^−1^ Co@N-PCNC within 12 min. The free radical quenching experiments were performed to reveal the nature of the reactive oxygen species radicals generated throughout the catalytic oxidation of CR. The effects of common inorganic anions and the water matrix on CR removal were studied. Moreover, the results of the kinetic study revealed the suitability of the pseudo-first-order and Langmuir–Hinshelwood kinetic models for illustrating CR degradation using the Co@N-PCNC/PMS system. Ultimately, the Co@N-PCNC displayed good operational stability, and after five cycles, the CR removal rate can still maintain over 90% after 12 min.

## Introduction

Dyes are widely utilized in massive amounts in various industries to impart color to a variety of products, such as paints, plastics, paper, leather, wood, textiles, etc.^1,2^. Among all dyes, azo dyes are the most widely used*,* accounting for nearly 50% of dyes consumed in the industry^3,4^. The vast prevalence of azo dyes is poisonous, mutagenic, and carcinogenic, besides being detrimental to the whole aquatic environment^5,6^. Unfortunately, substantial portions of the dyes are lost in the effluents during the production and dyeing processes^4,7,8^. Wastewater effluents coming out from dye-based industries need to be remediated efficiently before being released into nearby receiving waterways to evade environmental pollution and safeguard human health^9^. However, most of the azo dyes in use were found to be non-degradable and highly persistent and thus cannot be completely eliminated via conventional remediation approaches^10,11^. Thus, there is consistently a great need to develop efficacious treatment strategies to get rid of these recalcitrant contaminants from dye industry wastewater^9^.

Advanced oxidation processes (AOPs), including conventional Fenton, ozonation, peroxidation, persulfate oxidation, photooxidation, electrochemical oxidation, and their combination**,** have lately drawn an increasing amount of attention because of their competence in the fast elimination of several highly toxic and stubborn organic pollutants from wastewater^6,11,12^. These processes generate highly reactive oxidizing radicals, such as hydroxyl (·OH) and sulfate (SO_4_^**·**−^) radicals, from diverse peroxides that can directly oxidize organic contaminants into harmless small molecules, such as carbon dioxide and water^6,13^. Among various AOPs*,* growing attention has been directed to sulfate radical-based AOPs (SR-AOPs) during the last two decades owing to the high oxidation potential, prolonged life cycle, superior selectivity towards organic compounds, as well as broad pH suitability of the sulfate radical^9,12,14^. In general, the active SO_4_^•−^ can be created through the activation of persulfates, such as peroxydisulfate (PDS, S_2_O_8_^2−^) or peroxymonosulfate (PMS, HSO_5_^−^) [Eqs. (1) and (2)]^13^. Unlike PDS, PMS has an asymmetrical molecular structure, which makes its activation easier with lower energy intake, rendering SR-AOPs more economically and ecologically favorable^15–17^.

1 $${\text{S}}_{2} {\text{O}}_{{8}}^{2 - } \to 2{\text{SO}}_{{4}}^{ - }$$

2 $${\text{HSO}}_{{5}}^{ - } \to {\text{SO}}_{4}^{{{\mathbf{ \cdot }} - }} + \cdot {\text{OH}}$$

So far, diverse strategies have been proposed in the literature through the years to activate persulfates. Examples include heat treatment^13^, UV irradiation^18^, ultrasound^19^, catalysts (e.g., transition metals^17,20,21^, and carbon-based materials^22^). Indeed, heterogeneous catalytic activation by transition metals delivers numerous distinct benefits over other activation techniques in terms of high activation efficacy, simplistic operation, easy separation and recyclability, and low energy requirements^16,23^. So far, various transition metal-based heterogeneous catalysts have been scrutinized as potential catalysts for PMS activation^16,17,20,21,24^. Studies developed in recent years have ventured the superiority of Co-based catalysts over other transition metal-based catalysts for PMS activation^9,12,14,25–27^. Nevertheless, the widespread implantation of Co-based catalysts for PMS activation seems to be obstructed by their unstable performance due to severe metal particle aggregation/leaching during the remediation process^25^. Moreover, the leaching of the Co ions induces secondary contamination. The secondary pollution induced by the leached cobalt species is a true ecological challenge^28,29^. Cobalt is biologically toxic, which upon exposure may bring about solemn and sober menaces to mankind’s health, such as diarrhea, vomiting, cardiovascular disease, hyperglycemia, thyroid damage, and bone deformations^30–33^. Cobalt (II) compounds have been reported to cause DNA destruction, genetic alterations, and aneuploidy in living cells^34^. So, profuse research endeavors have been directed to develop novel cobalt-based heterogeneous catalysts for PMS activation with both high activity and robustness against metal leaching/agglomeration.

An effective tactic to address the above-mentioned shortcomings is to incorporate cobalt particles into proper porous, robust support materials^25,27,35,36^. Please note that the immobilization of the cobalt species over solid supports results in a catalyst not only with higher immunity against leaching during PMS-based AOP but also with higher oxidation performance as a result of the unique properties emanating from the interaction between the cobalt species and the support surface^37^. To date, various porous materials have been scrutinized as supports for Co species in PMS activation, including zeolites, alumina, titania, mesoporous silica, and carbon-based materials^9,15,23^. Of these, porous carbon-based materials, like carbon black^25^, biochar^36^, heteroatom-doped porous carbon^37^, reduced graphene oxide^27^, carbon nanotubes^26,35^, have occupied a special place because of their extraordinary properties, including vast availability, uncomplicated synthesis, eco-friendly character, exceptional chemical and mechanical stabilities, good electrical conductivity, ample surface functionalities, high surface area and tunable porosity^12,23,38^. It is worth mentioning that porous carbon-based materials usually possess a high adsorption capacity for various organic contaminants, which would lead to enhanced enrichment of the organic contaminants around the active sites on the surface of the catalyst, thereby resulting in more efficient degradation of pollutants^36^. Moreover, the carbon support can evidently facilitate the electron transfer during pollutant oxidation by the electron tunneling effect at the interface of Co particles and the carbon support and thus effectively enhance the PMS-based degradation^39^. Furthermore, the catalytic performance of carbon-supported cobalt catalysts in PMS activation can be substantially boosted by introducing non-metal heteroatoms, such as N, S, B, Si, and I, in the carbon matrix because of the improved electronic transfer property and the probable synergetic interaction between Co and the heteroatoms^23,38,40,41^. Unfortunately, most of the manufacturing strategies employed to obtain porous Co-embedded heteroatom-doped carbon catalysts are complex, costly, and thus restricted to the laboratory scale and cannot fulfill the necessities of commercial production^15^. Consequently, it is extremely crucial to develop facile and scalable preparation strategies for synthesizing cobalt and nitrogen co-doped carbon-based catalysts with exceptional catalytic activity and stability for PMS activation and azo dye degradation.

Metal–organic frameworks-mediated synthesis (MOFMS) has been proven to be a simple and effective technique for synthesizing various composites of nanoparticles encapsulated in a porous carbon matrix. In this route, MOFs act as both precursors and morphological templates to create nano-composites through pyrolytic treatment at high temperatures under a flow of inert gas. These MOF-derived nanomaterials often display distinct advantages like elevated surface area, tunable porosity, excellent chemical and mechanical stabilities, tailorable morphologies, and controllable functionalities, which endow them with exceptional performance in catalysis, gas storage and separation**,** magnetism, sensing, drug delivery, energy storage, and others^42,43^. ZIF-67 is one of the most widely studied MOFs built up from Co^2+^ cations coordinated to nitrogen-rich 2-methylimidazole organic ligands^43,44^. ZIF-67 is deemed an attractive precursor to fabricate Co/nitrogen-doped carbon-based catalysts through MOFMS owing to its inexpensive and straightforward synthetic procedure, large specific surface area, tailored morphology and porosity, and high content of carbon and nitrogen elements^45–48^. During high-temperature pyrolysis in an inert environment, the 2-methylimidazole organic ligands in ZIF-67 could be readily converted into a porous carbonaceous matrix with an ample amount of nitrogen, and simultaneously, the cobalt metal nodes within the framework are reduced by the N-rich carbon matrix to form highly dispersed and encapsulated metallic Co nanoparticles^44,49^. Over the previous few years, research activity has been devoted to evaluating ZIF-67-derived nitrogen**-**doped carbon**-**supported cobalt materials as potential heterogeneous catalysts for PMS activation^50,51^.

**Figure 1:**
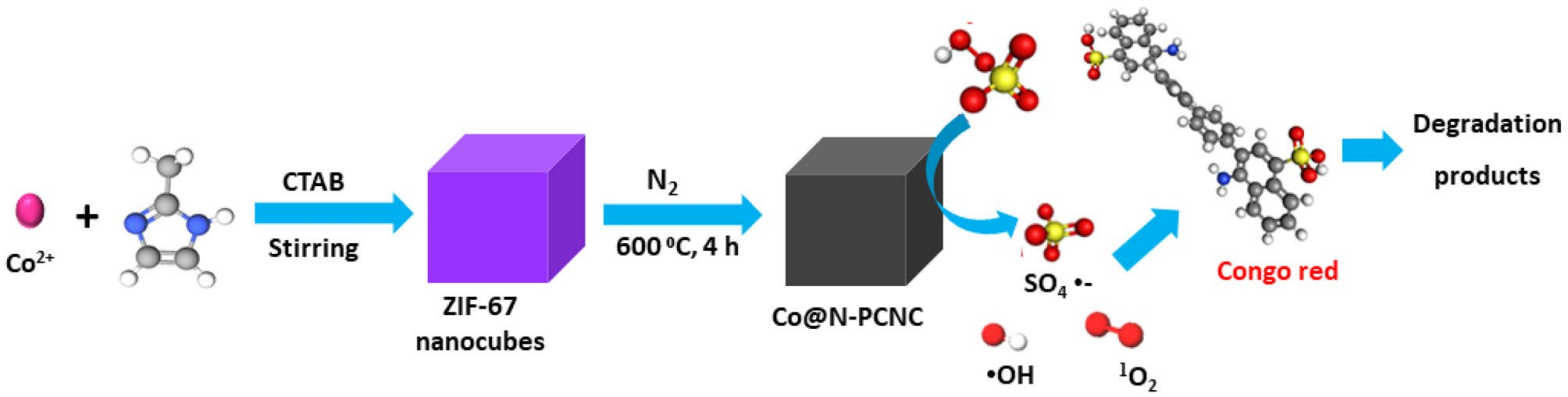
Degradation of Congo Red dye by peroxymonosulfate with ZIF-67-derived Co/N-doped porous carbon nanotube.

Herein, the MOFMS strategy was successfully adopted to fabricate Co^0^/N-doped porous carbon nanocubes (Co@N-PCNC) hybrid by pyrolysis of ZIF-67. The fabricated Co@N-PCNC was adopted as a catalyst for activating PMS to the degradation of Congo red dye (CR) (Fig. 1). The main parameters affecting the catalytic degradation process, including catalyst amount, PSM dose, CR concentration, and common inorganic anions in water matrices, were scrutinized. The recyclability and operational stability of the catalyst during repetitive usage in PMS-assisted degradation of CR were studied as well. Furthermore, radicle quenching tests were carried out to identify the prevailing radical species in the PMS/Co@N-PCNC systems. Interestingly, Co@N-PCNC exhibited superior catalytic performance for PMS activation to degrade CR dye compared with other formerly reported catalysts.

## Experimental

### Synthesis of ZIF-67 nano-cubes

All chemicals were purchased and used without additional purification. ZIF-67 with cubic morphology was synthesized according to a literature-reported recipe with a slight modification^52^. In a typical synthesis, 5 mmol of cobalt nitrate hexahydrate was dissolved in CTAB aqueous solution (6.68 mM) and stirred at ambient temperature for 30 min to form solution A. After that, 9.08 mmol of 2-methylimidazole was dissolved in deionized water to form solution B. Then, solution A was poured rapidly into solution B under constant agitation. The solution color immediately turned purple because of the fast formation of ZIF-67 nanocrystals. After stirring for 20 min at room temperature, the purple precipitate was filtered off and washed consecutively with water and ethanol before being eventually vacuum-dried at 60 °C for 12 h.

### Synthesis of porous Co@N-PCNC

The ZIF-67 nanocubes were carbonized in a tube furnace at 600 °C for 4 h with a heating rate of 2 °C min^−1^, under flowing nitrogen. Any cobalt nanoparticles not firmly bound to the carbon matrix were eliminated by soaking the as-obtained carbonization product into a 2.0 M H_2_SO_4_ aqueous solution at ambient temperature for 24 h. The resulting Co@N-PCNC nanocubes were harvested by filtration, thoroughly rinsed with deionized water to neutrality, and then oven-dried at 70 °C for 12 h.

### Material characterizations

Information concerning the crystal structures of the synthesized samples was acquired by powder XRD (Bruker AXS D8 diffractometer) with Cu K*α* radiation (*λ* = 1.5406 Å). Thermogravimetric analysis (TGA) was conducted under constant nitrogen flow on a TA thermal analyzer at a rate of 15 °C/min from 25 to 800 °C. The FTIR spectra were recorded using an Agilent FTIR spectrometer with KBr. The N_2_ adsorption–desorption isotherms were collected using a Quanta Chrome Nova surface area and pore size analyzer at − 196 °C. The specific surface area of the samples was calculated by the BET method, and the total pore volume was determined from the volume of N_2_ taken up by the samples at P/P^0^ = 0.95. Besides, the BJH method was used to calculate the pore size distribution of the samples using the desorption branch of the isotherm. The surface morphologies of the samples were scrutinized with E-SEM (Thermo Scientific Quattro S). The chemical compositions and mapping images of the samples were determined by EDX associated with the E-SEM instrument. The XPS spectra were recorded on a Thermo-Fisher Scientific spectrometer with monochromatic X-ray Al Kα radiation. The total organic carbon (TOC) was measured on Analytic Jena multi N/C 2100S TOC analyzer.

### Catalytic activity tests

In each degradation experiment, 200 mL CR (50 mg L^−1^) aqueous solution and a particular amount of the catalyst were placed in a 250 mL beaker. After that, the resulting solution was sonicated in the dark in an ultrasonic bath for 10 min at ambient conditions to guarantee uniform dispersion of the catalyst. Then, a predesigned dosage of PMS was added to the reaction mixture, and the degradation started. A 3 mL aliquot was sampled at predetermined time intervals, filtered using a syringe filter (0.2 μm), and analyzed with an ultraviolet–visible spectrophotometer (JASCO, V-750 UV–visible Spectro-photometer) at 500 nm for the residual concentration of CR. Experiments were carried out to dissect the impact of catalyst amount, PSM dose, CR concentration, and inorganic anions on CR removal efficiency. To assess the reusability of Co@N-PCNC, the catalyst, after completion of the degradation, was magnetically isolated from the reaction mixture, rinsed with water and ethanol, and vacuum-dried at 60 °C for 1 h before being subjected to a second run of the reaction process with fresh reactants under identical conditions to the first run. The regeneration and reusability of the used catalyst were repeated three times following the procedure explained above. Additionally, to determine the prevailing radical species formed in the PMS/Co@N-PCNC systems, quenching investigations were executed employing the same procedure for the degradation experiment, only adding 1000 mM methanol (MeOH) or 1000 mM tertbutyl alcohol (TBA), as the scavenging agents**,** to the reaction mixture.

## Results and discussions

### Catalyst characterization

The adopted procedure in this work for the synthesis of Co@N-PCNC involves, firstly, the synthesis of cubical ZIF-67 via the precipitation method at room temperature (~ 25 °C) in the presence of CTAB. ZIF-67 is a well-studied Co-containing MOF composed of Co^2+^ cations as the metal nodes connected by 2-methyl imidazolate linkers. The obtained ZIF-67 nanocubes were then subjected to direct carbonization at 600 °C under an N_2_ atmosphere to derive metallic Co nanoparticles well-distributed in porous carbon nanocubes (Co@N-PCNC). During the inert pyrolysis of the ZIF-67 nanocubes, the Co ions were converted into ultra-small metallic Co nanoparticles. Meanwhile, the 2-methylimidazole organic linker was carbonized to porous nitrogen-rich carbon nanocubes encapsulating the Co nanoparticles.

**Figure 2:**
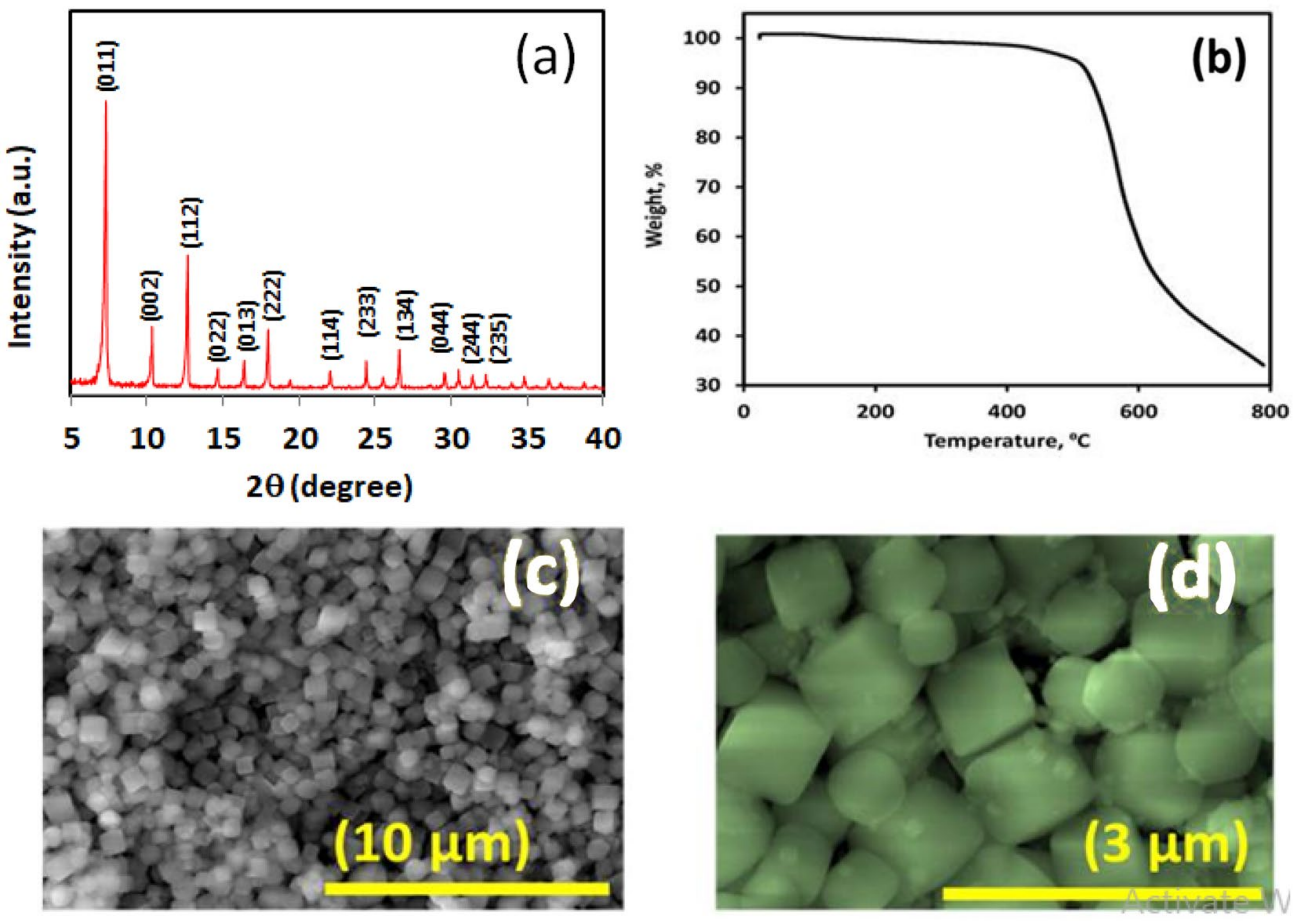
XRD pattern (**a**), TGA curve (**b**), and FE-SEM images (**c**,**d**) of ZIF-67 nanocubes.

The XRD of ZIF-67 is manifested in Fig. 2a. The PXRD pattern of the as-synthesized ZIF-67 sample perfectly agreed with those reported previously in the literature (JCPDS card no: 62-1030)^53^, suggesting the successful preparation of the pure ZIF-67 phase. The peaks, which appeared at 7.2°, 10.4°, 12.7°, 14.7°, 16.4°, 18°, 24.5°, 26.5°, 29.6°, 31.3°, and 32.5°, are related to the (011), (002), (112), (022), (013), (222), (233), (134), (044), (244), and (235) lattice planes, respectively^54^. No other diffraction lines were detected, demonstrating the high purity of the obtained ZIF-67 precursor.

The TGA thermogram of ZIF-67 reveals only one thermal event (Fig. 2b), as reported formerly^55^, at above ~ 500 °C, which can be attributed to the thermal decomposition of the ZIF-67 framework. From the TGA curve, it is evident that the ZIF-67 nanocubes have high stability and begin to decompose at above ~ 500 °C. In the sight of this result, a temperature of 600 °C was chosen as the annealing temperature for the transformation of ZIF-67 into Co@N-PCNC in N_2_.

The FTIR spectrum of pristine ZIF-67, in Fig. S1, manifests bands at around 685.8 and 752.9 cm^−1^, corresponding to the out-of-plane deformation vibrations of the imidazole ring^56^. Besides, the bending and stretching vibrations of the C–N bond in the imidazole ring may account for the absorption bands that emerged at 991.5 and 1140.6 cm^−1^, respectively^57^. The absorption due to the stretching modes of the C=N was typically positioned^57^ at 1565.4 cm^−1^, whereas the peak at 1304.6 is likely to arise from C=C stretching^58^. The prominent band at 1416.4 cm^−1^ points to the bending vibration of the CH_3_ group^58^, while the two bands at 2936 and 3145 cm^−1^ could be linked to the stretches of the aromatic and aliphatic C–H of the imidazole ring, respectively^59^. The band at 3616 cm^−1^ belongs to the O–H stretching vibration^58^.

The porous properties of the ZIF-67 precursor were dissected by means of N_2_ physisorption, and the resulting isotherm is manifested in Fig. S2. As is depicted in this figure, the pristine ZIF-67 yielded a type I isotherm featured by an abrupt rise in the amount of N_2_ adsorbed in the low relative pressure (P/P^o^) region of the isotherm, demonstrating its microporous character. In addition, the parent MOF material exhibited a high BET surface area and large pore volume of 1547.5 m^2^ g^−1^ and 0.68 cm^3^ g^−1^, respectively. It is worth mentioning that these values are in the range of the values reported in the literature^52^, further verifying the formation of the ZIF-67 structure.

The morphology of the fabricated ZIF-67 sample was investigated by FESEM. The FESEM image in Fig. 2c,d shows that the parent ZIF-67 has a clear cubic morphology with a smooth surface and particle sizes ranging from 200 to 700 nm.

**Figure 3:**
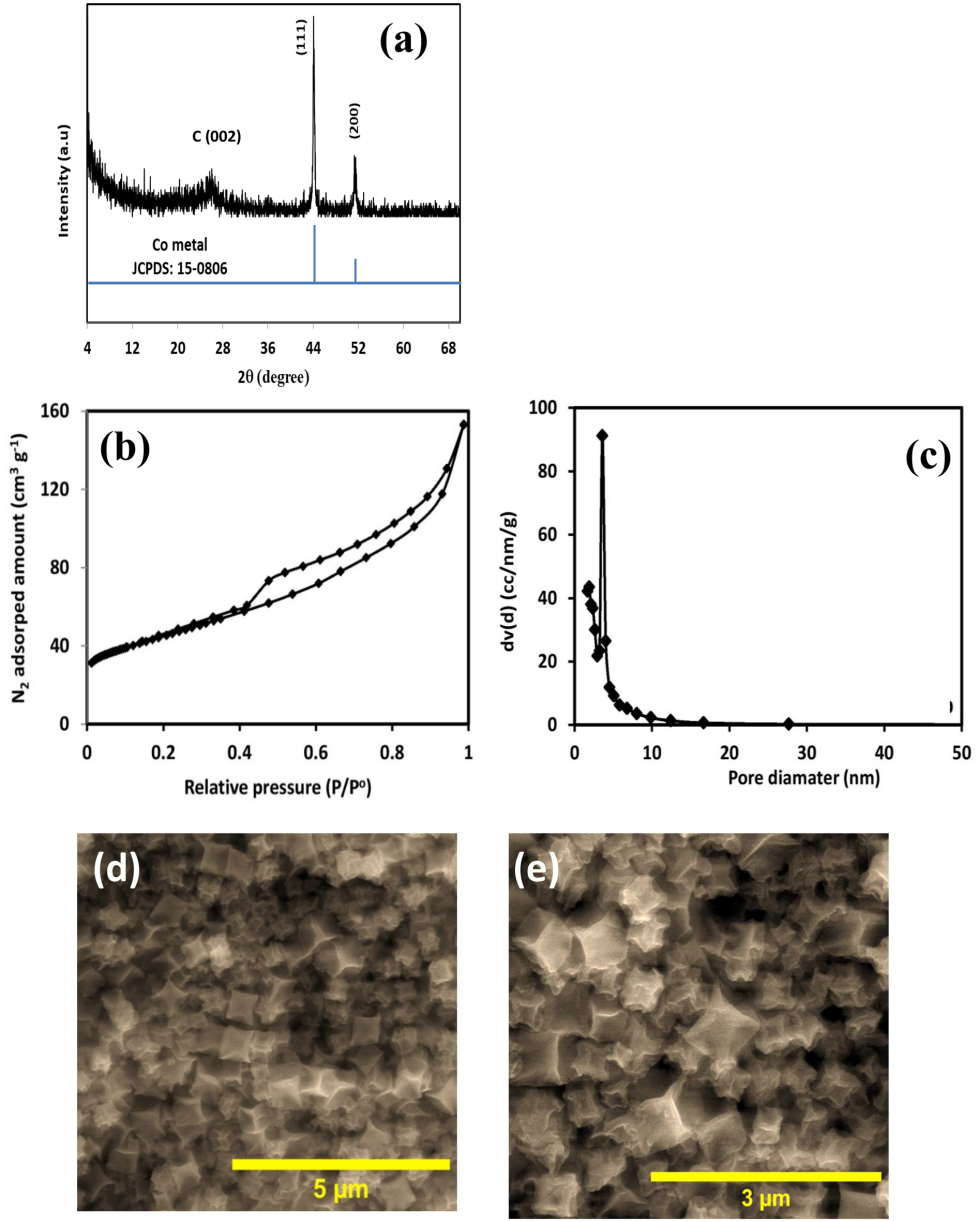
XRD pattern (**a**), N_2_ physisorption isotherm (**b**), pore size distribution curve (**c**), and FE-SEM images at different magnifications (**d**,**e**) of Co@N-PCNC.

To scrutinize the crystal structure of the obtained Co@N-PCNC material, XRD analysis was executed. In the XRD profile of Co@N-PCNC (Fig. 3a), the diffraction line positioned at 2θ of 26.3° can be assigned to the (002) diffraction facets of carbon, derived from the decomposition of the organic linker, while those at 2θ of about 44.7°, and 51.9° can be assigned to the (111), and (200) diffraction facets of metallic cobalt (JCPDS: 15-0806), respectively.

Figure 3b illustrates the N_2_ adsorption–desorption isotherm curve (Fig. 3b) and the corresponding desorption BJH pore size distribution plot (Fig. 3c) of the Co@N-PCNC sample. As shown in Fig. 3b, the isotherm of the carbonized material is obviously different from that of ZIF-67. The Co@N-PCNC catalyst manifests a typical type IV isotherm with an obvious hysteresis loop in the range of ca. 0.5–1.0 P/P^o^ as a result of the capillary condensation, pointing out the mesoporous nature of the as-obtained Co@N-PCNC material. This indicates that mesopores are formed accompanying the collapse of ordered micropores. The carbonized material has a specific surface area and pore volume of 155.32 m^2^ g^−1^ and 0.23 cm^3^ g^−1^, respectively. It is worth mentioning that the specific surface area of Co@N-PCNC is far lower than the parent ZIF-67 (1547 m^2^ g^−1^). The decrease in surface area can be attributed to the inevitable collapse of the well-defined microporous structure of the ZIF-67 skeleton caused by the decomposition of the imidazole ligands with the release of CO_2_ and CO during the slow thermolysis process, resulting in the creation of a new mesoporous structure^60–62^. The Co@N-PCNC specimen exhibited a considerably larger mesoporous volume as compared to the parent ZIF-67 (0.22 vs. 0.08 cm^3^ g^−1^). In contrast, the micropore volume of the carbonized sample (0.01 cm^3^ g^−1^) is less than that of the ZIF-67 sample (0.6 cm^3^ g^−1^). This change in the porous properties demonstrates that the original microporous structure of ZIF-67 was converted into a mesoporous structure after the annealing treatment^63^. The pore size distributions obtained from the isotherms (Fig. 3c) further indicate the mesoporous feature of the sample, with most pores being centered at 3–4 nm. This mesoporous structure ensures the full exposure of the active sites of the catalyst and effectively enhances the diffusion and mass transfer of the reactants and products, resulting in efficient catalysis^64^.

As can be noticed in the SEM images (Fig. 3d,e), Co@N-PCNC still retains the cubic outline of the parent ZIF-67 but shrinks. Nevertheless, the surface of Co@N-PCNC is rougher than that of the parent ZIF-67, which could be attributed to the decomposition of organic linkers in the parent ZIF-67, yielding additional mesoporosity. The SEM–EDS and Mapping data (Fig. S3 and Table S1) revealed that Co@N-PCNC contained carbon, oxygen, nitrogen, and cobalt, and they were well spread across the selected region of the sample.

**Figure 4:**
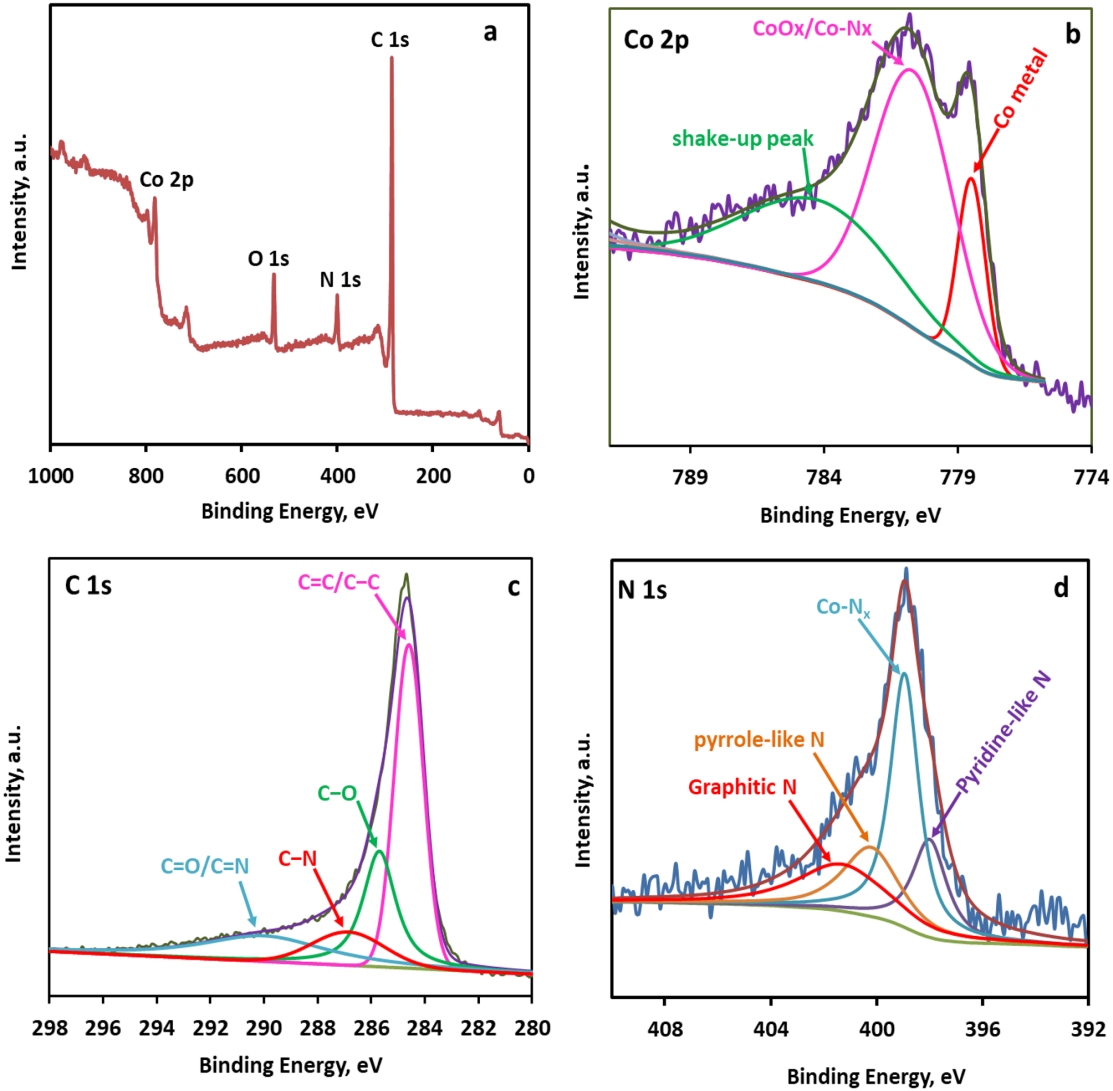
XPS spectra of Co@N-PCNC. (**a**) Survey scan, (**b**) Co 2p_3/2_ spectrum, (**c**) C1s spectrum, and (**d**) N 1s spectrum.

XPS was executed to scrutinize the elemental composition and oxidation states of the surface species on the Co@N-PCNC catalyst. The XPS survey spectra of Co@N-PCNC (Fig. 4a) confirmed the existence of Co, O, N, and C elements. The presence of oxygen in Co@N-PCNC was probably a result of the absorption of oxygen from the atmosphere^52^. The deconvolution of the XPS Co 2p_3/2_ spectra (Fig. 4b) yields four distinctly defined peaks positioned at binding energies of 778.68, 781, and 784.4 eV, which corresponded to Co metal, CoO_x_ (Co^2+^, and Co^3+^)/Co-N_x_, and shake-up peak, respectively^52,65^. Although the XRD analysis showed that cobalt species in the catalyst exist as metallic cobalt only confirmed the sole existence of zero-valent cobalt metallic cobalt in the catalyst, the XPS analysis showed the presence of cobalt oxide in addition to the metallic cobalt. Similar observations were reported in previous works^52^. Co^2+^ and Co^3+^ species are formed by the unavoidable surface oxidation of the Co NPs by atmospheric oxygen. Upon the deconvolution of the C 1s spectrum, four distinct peaks emerged at binding energies of 284.78, 286, 287.38, and 290.6 eV and were attributed to C=C/C–C, C–O, C–N, and C=O or C=N functionalities, respectively (Fig. 4c)^66,67^. Figure 4d manifests the deconvoluted N 1s spectra, which depict four prevalent peaks located at approximately 398, 398.94, 400.2, and 401.3 eV binding energies. The prominent peak at 398.94 eV is designated to N atoms in Co-N_x_ structural moieties; meanwhile, pyridine-like nitrogen, pyrrole-like nitrogen, and graphitic nitrogen are responsible for other peaks at 398, 400.2, and 401.3 eV binding energies, respectively^66,68^. The Co-Nx structural moieties were presumably formed as a result of the interaction between Co particles and the neighboring N atoms^69^.

### Efficient activation of PMS by Co@N-C for CR removal

**Figure 5:**
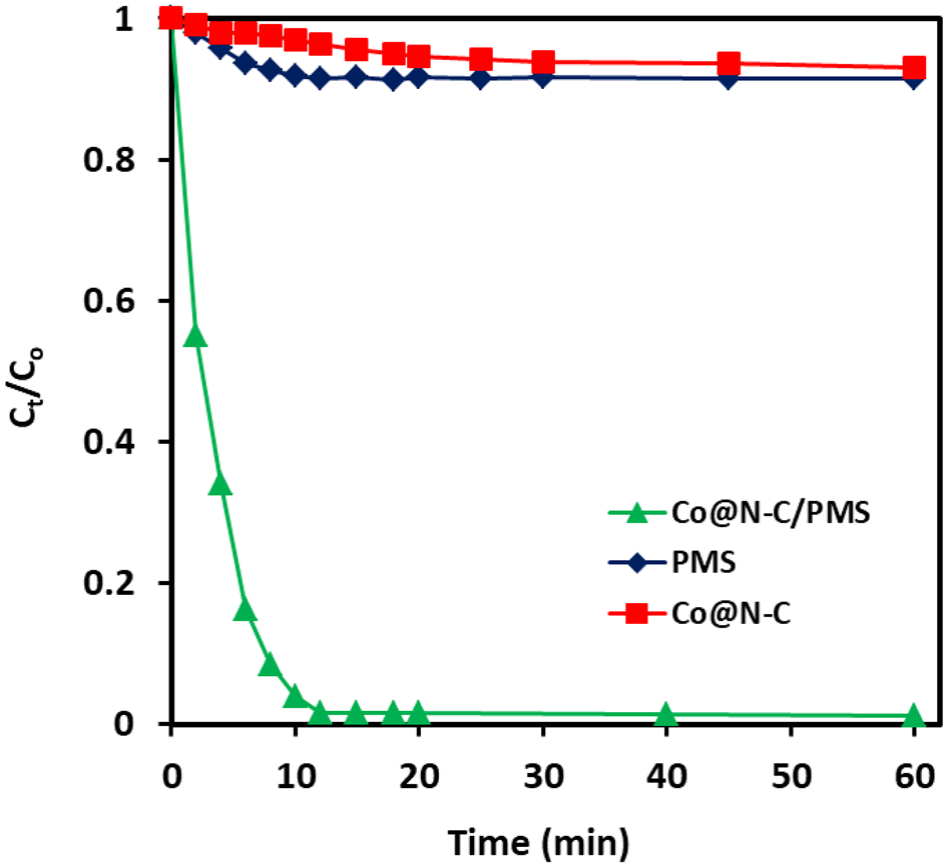
The removal efficiency of CR at different systems.

The degradation of CR in batch mode was chosen as a model reaction to appraise the catalytic performance of the synthesized Co@N-PCNC. The catalytic performance of the as-obtained Co@N-PCNC catalyst is depicted in Fig. 5. Please note that all the degradation experiments were executed at the natural pH of the solution. Figure 5 compares the change in the degree of CR degradation (in terms of C_t_/C_o_) as a function of time during the oxidation process in the presence of Co@N-PCNC alone, PMS alone, or Co@N-PCNC/PMS, respectively. The results manifested that only 8.2% of the CR dye was degraded within 60 min in the absence of the catalyst, designating that PMS alone is incapable of oxidizing CR to a substantial extent, and reaction can occur only in the presence of a proper catalyst. Additionally, in the absence of PMS, no noticeable change in CR dye concentration was discerned over time, and only 6.9% of the initial dye concentration was removed within 60 min, intimating that the adsorption contribution to the CR removal can be disregarded. Nonetheless, from Fig. 5, one can see that when Co@N-PCNC catalysts (75 mg L^−1^) were used concomitantly with PMS (0.033 mM), a high degree of CR degradation of 98.45% was achieved after the elapse of 12 min at room temperature. This substantial enhancement in CR degradation efficiency originated from the activation of PMS by the cobalt-based catalysts, which resulted in the generation of highly reactive oxidizing species, which have high oxidation potential and can efficiently degrade CR molecules^70,71^.

The degree of mineralization of the CR dye in the Co@N-C/PMS process can be assessed by monitoring the depletion in TOC removal efficiency as a function of time, as manifested in Fig. S4. From the figure, it is evident that after the elapse of 12 min of reaction, about 44.46% of the TOC removal efficiency of CR was obtained. In other words, the TOC removal was less than the CR degradation efficiency. This signifies that CR dye was only partially mineralized, and various organic intermediates were created in the solution during the course of the oxidation of CR. It is worth mentioning that TOC removal can be further improved by prolonging the oxidation duration.

#### Effects of operational conditions on CR degradation in Co@N-C/PMS system

**Figure 6:**
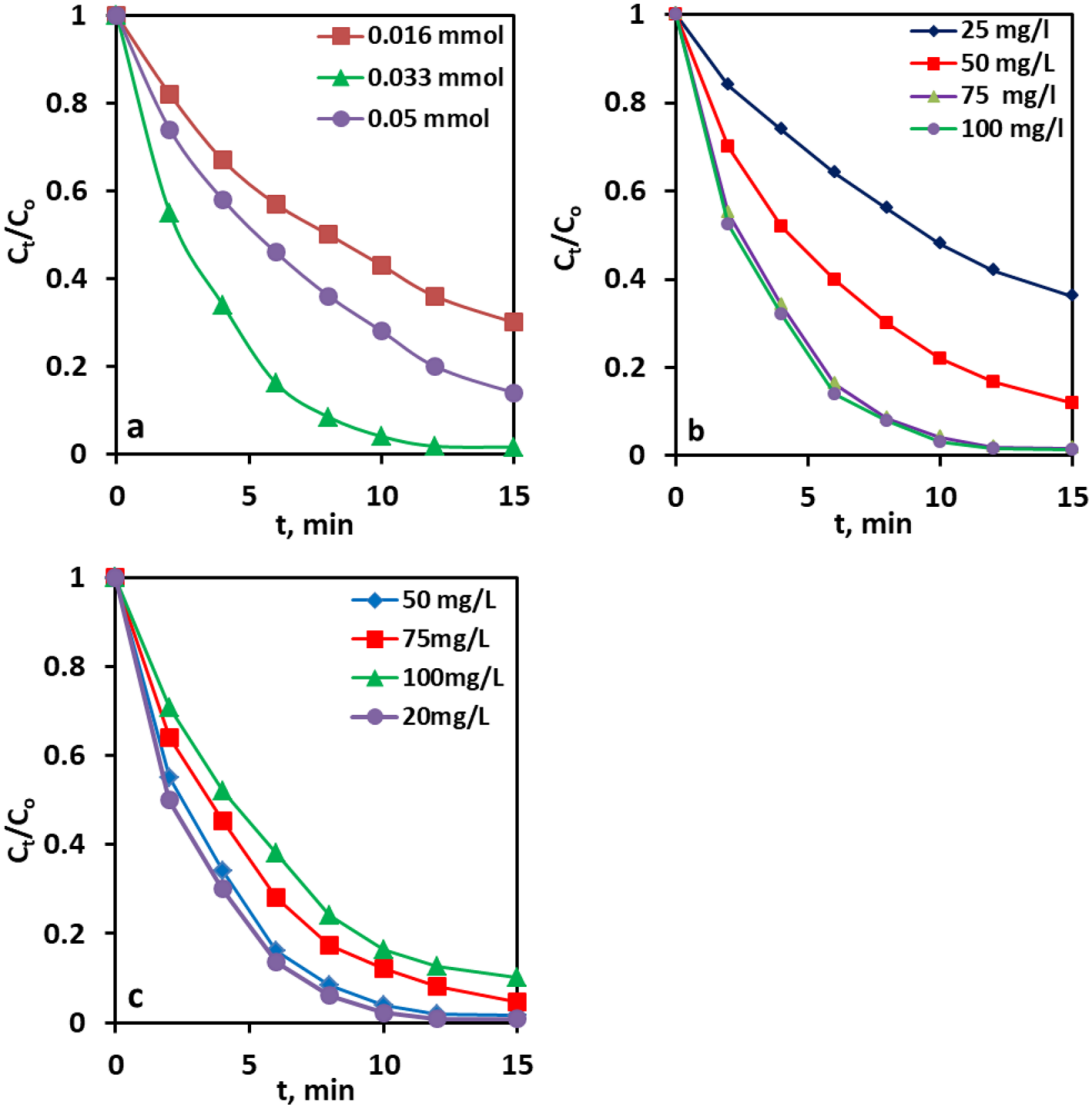
The effects of PMS concentration (**a**), catalyst dosage (**b**), CR dye concentration (**c**) on CR removal in Co@N-PCNC/ PMS system.

The impact of the PMS concentration on the catalytic degradation of CR efficiency in the Co@N-PCNC /PMS system was scrutinized by diversifying the PMS dosage from 0.016 to 0.05 mM; the experimental results are manifested in Fig. 6a. Clearly, the PMS concentration had a powerful influence on the degradation efficiency. The degradation efficiency increased with the increase in PMS concentration up to 0.033 mM and then decreased beyond this value. The increase in PMS concentration led to the formation of more reactive radicals, boosting the degradation efficiency of CR. Nevertheless, a PMS concentration of more than 0.033 mM was not beneficial to the degradation efficiency because the highly reactive SO^4−**·**^ and ·OH radicles, instead of being employed in the oxidative degradation of CR dye, would react with the surplus PMS, giving rise to the less active peroxomonosulfate radicals, SO^5−**·**^, resulting in a decrease in CR degradation efficiency (Eqs. (3) and (4))^72–74^. Moreover, excess SO^4−**·**^ and SO^5−**·**^ would also be involved in self-quenching reactions, as depicted in Eqs. (5) and (6), thus lowering the quantity of **c**atalytically active radicals^74,75^. Based on the results, 0.03 mM was chosen as the optimal PMS concentration to accomplish fast CR degradation.

3 $${\text{SO}}_{{4}}^{{ - {\mathbf{ \cdot }}}} + {\text{HSO}}_{5}^{ - } \to {\text{SO}}_{{5}}^{{ - {\mathbf{ \cdot }}}} + {\text{SO}}_{{4}}^{2 - } + {\text{H}}^{ + }$$

4 $${\text{ OH}}^{ \cdot } + {\text{HSO}}_{{5}}^{ - } \to {\text{SO}}_{{5}}^{{ - {\mathbf{ \cdot }}}} + {\text{H}}_{2} {\text{O}}$$

5 $${\text{SO}}_{{4}}^{{ - {\mathbf{ \cdot }}}} + {\text{SO}}_{{4}}^{{ - {\mathbf{ \cdot }}}} \to {\text{S}}_{2} {\text{O}}_{{8}}^{2 - }$$

6 $${\text{SO}}_{5}^{{ - {\mathbf{ \cdot }}}} + {\text{SO}}_{5}^{{ - {\mathbf{ \cdot }}}} \to {\text{S}}_{2} {\text{O}}_{8}^{2 - } + {\text{O}}_{2}$$

Catalyst amount is another crucial parameter that affects the degradation efficiency. To optimize the catalyst amount, a set of experiments with varying amounts of Co@N-PCNC between 25 and 100 mg L^−1^ were conducted at 25 °C and 50 mg L^−1^ CR, and the results are manifested in Fig. 6b. A high removal efficiency of 66.2% was achieved even at a low Co@N-PCNC concentration of 25 mg L^−1^ within 20 min, which further affirmed the high catalytic efficiency of the catalyst. An evident increase in CR degradation efficiency, from 66.2 to 94.2%, in 20 min was discerned when the Co@N-PCNC concentration was raised from 25 to 50 mg L^−1^. With the further increment in the Co@N-PCNC concentration to 75 mg L^−1^, about 98.2% of CR was removed after the elapse of 12 min. The improvement can be assigned to the increased number of available catalytic active sites for activating PMS. A further increase in the catalyst amount to 100 mg L^−1^ provoked almost no significant enhancement in the degradation efficiency. Keeping the economic aspect in mind, 75 mg L^−1^ catalyst was selected as the optimal economical and efficient catalyst amount for CR degradation, and thereby, will be used in the continuation of this study.

The degradation efficiency was also assessed as a function of CR concentration in the range of 20–100 mg L^−1^, and the results are shown in Fig. 5c. As expected, the degradation efficiency has experienced a decrease with the increase in CR concentration. As shown in Fig. 6c, when the concentration of CR was 100 mg L^−1^, the degradation efficiency declined to 87.5% within 12 min. This result is likely due to insufficient active sites^76^.

A comparison of the performance of Co@N-PCNC for the PMS**-**assisted degradation of CR with various heterogeneous catalysts in the literature is compiled in Table 1. As demonstrated in Table 1, most reported catalysts offered high CR degradation efficiencies. However, compared with the mentioned catalysts, the as-synthesized Co@N-PCNC catalyst offers some benefits, including high CR degradation efficiency in a relatively short duration employing low catalyst and PMS doses with no need for pH adjustment, leading to an economical and sustainable remediation process. Ultimately, it deserves noting that the PMS activation process adopting Co@N-PCNC requires no extra light or ultrasonic assistance.

**Table 1:** Comparison of Co@N-PCNC with other heterogeneous catalysts for the oxidative removal of CR with PMS.

	Catalyst	Reaction conditions						Degradation	References
		[Dye], mg L^−1^	[Catalyst], g L^−1^	[PMS], mM	pH		T, ℃		
1	CNT@ZIF-800	50	0.05	1	Natural		25	98.50% (5 min)	^69^
2	CoFe/CoFe_2_O_4_	60	0.05	9.8		7.0	20	97.00% (3 min)	^77^
3	CoFe_2_O_4_@MC	20	0.05	0.3		7.0	25	93.23% (30 min)	^78^
4	CoFe_2_O_4_ @MXene	30	0.25	0.3		9	25	98.9 (30 min)	^79^
5	CoFeNi-LDH	20	0.2	0.001	7		25	100.00% (6 min)	^80^
6	5S@Fe-500	50	0.6	3.9	Natural		30	98.90 (10 min)	^69^
7	C@Co_3_O_4_-Q5	20	0.2	1	Natural		25	95.00% (40 min)	^81^
8	C-Fe	50	1	6.6	9		25	86.00%(min)	^82^
9	FeCo-BDC	20	0.02	0.25	4.68		25	95.00% (5 min)	^83^
10	SCMG	20	One piece	0.1	7		25	95.60% (2 min)	^84^
11	Co@NCNTs-600	20	0.6	1	7		25	98.10% (8 min)	^85^
12	Fe-NC	20	0.025	0.33	Natural		25	100% (50 min)	^86^
13	Fe_3_O_4_-CoCO_3_/rGO	20	0.040	3.28	7		25	≈ 60.00% (3 min)	^87^
14	Co@N-PCNC	50	0.075	0.033	Natural		25	98.48 (12 min)	This study

CoFe_2_O_4_@MC: mesoporous carbon-anchored cobalt ferrite nanocomposites.

5S@Fe-500: sludge-based biochar material loaded with nano-Fe_3_O_4_.

C@Co_3_O_4_-Q5: Co_3_O_4_ quantum dots (QDs) decorated hierarchical C@Co_3_O_4_.

C-Fe: Chemogenic Fe-NPs.

FeCo-BDC : FeCo-based bimetallic metal organic framework (MOF) nanosheet.

SCMG: 3D Co/Mo co-catalyzed graphene sponges.

Fe-NC: isolated Fe atoms coordinated with N species embedded in carbon polyhedrons.

#### Effect of Co-existing anions and water matrix

**Figure 7:**
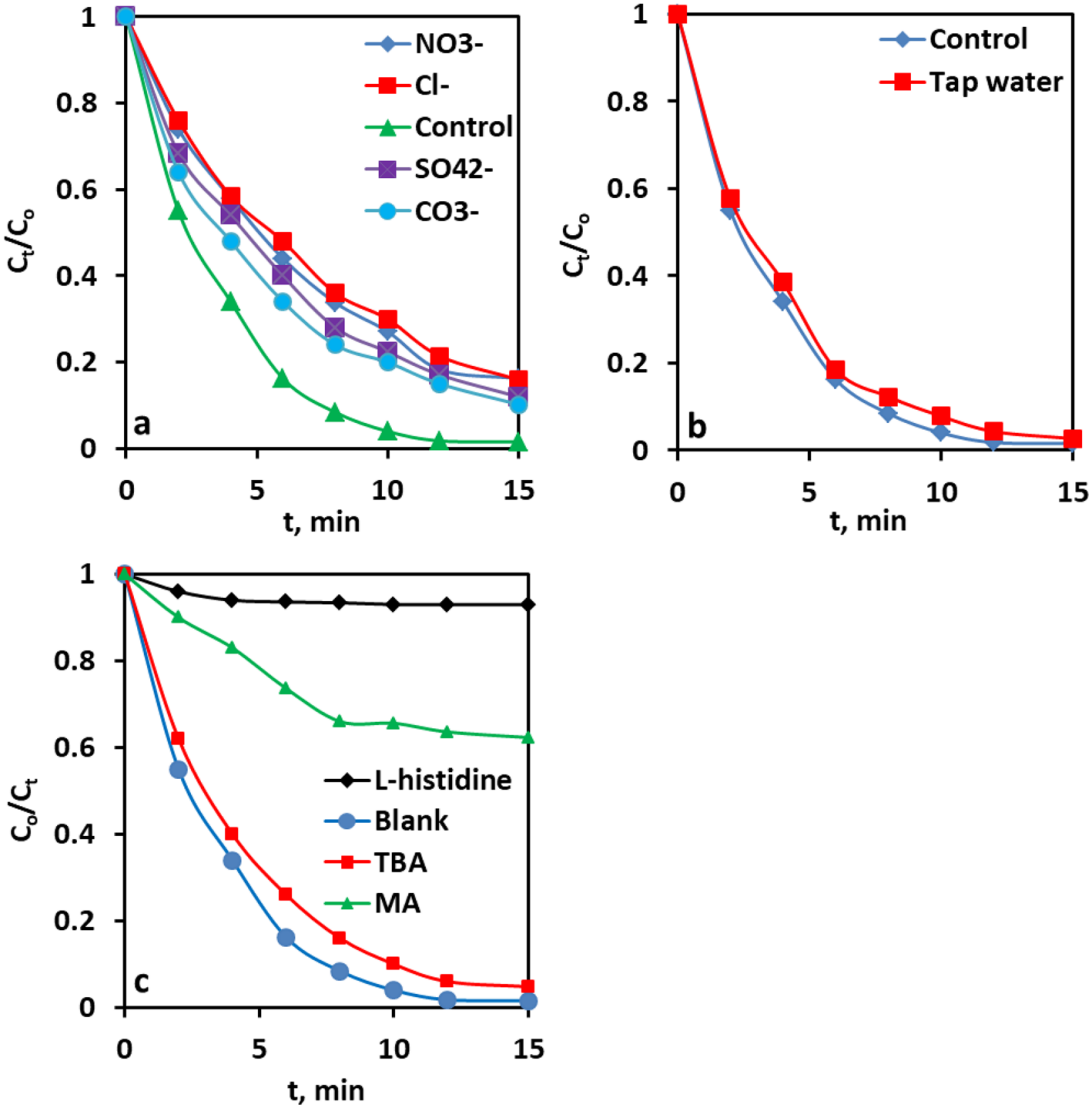
The effects of common anions (**a**), and water matrix (**b**), free radical scavengers (**c**) on CR removal in Co@N-PCNC/PMS system.

To appraise the realistic applicability of the Co@N-PCNC /PMS system, the dye degradation experiments were also conducted in the presence of nitrate, chloride, sulfate, and bicarbonate anions (at 10 mmol L^−1^), which are almost inevitably present in actual water matrices. The outcomes of these experiments are manifested in Fig. 7a. As shown in Fig. 7a, the addition of Cl^−^, SO_4_^2−^, CO_3_^2−^, and NO_3_^−^ negatively impacted CR degradation efficiency. The inhibitory impact of Cl^−^, SO_4_^2−^, CO_3_^2−^, and NO_3_^−^ was mainly because these anions could negatively consume the highly reactive OH^**·**^ and SO_4_^**·**^ radicles to give rise to less reactive radicals, resulting in a decrease in CR degradation efficiency, in line with Eqs. (7–13)^9^. Besides, some inorganic anions may also competitively occupy the active sites on the catalyst surface, thereby restricting the catalytic degradation of the target organic pollutants^9,88^. Importantly, in the presence of Cl^−^, SO_4_^2−^, CO_3_^2−^, and NO_3_^−^ anions, Co@N-PCNC can still achieve satisfactory CR degradation efficiency of 78.6%, 82.9%, 85.45, and 81.7% in 12 min, respectively, thereby demonstrating that the Co@N-C/PMS system can effectively resist the interference of the general inorganic anions.

7 $${\text{Cl}} \cdot + {\text{SO}}_{{4}}^{ \cdot } \to {\text{SO}}_{{4}}^{2 - } + {\text{Cl}} \cdot$$

8 $${\text{Cl}} \cdot + {\text{OH}} \cdot \to {\text{ClOH}}^{{{\mathbf{ \cdot }} - }}$$

9 $${\text{Cl}}^{ - } + {\text{Cl}} \cdot \to {\text{Cl}}_{{2}}^{{{\mathbf{ \cdot }} - }}$$

10 $${\text{SO}}_{{4}}^{{{\mathbf{ \cdot }} - }} + {\text{SO}}_{{4}}^{2 - } \to {\text{SO}}_{{4}}^{2 - } + {\text{SO}}_{{4}}^{{{\mathbf{ \cdot }} - }}$$

11 $${\text{OH}} \cdot + {\text{ SO}}_{{4}}^{2 - } \to {\text{SO}}_{{4}}^{{{\mathbf{ \cdot }} - }} + {\text{OH}}^{ - }$$

12 $${\text{SO}}_{{4}}^{{{\mathbf{ \cdot }} - }} + {\text{ CO}}_{{3}}^{2 - } \to {\text{SO}}_{{4}}^{2 - } + {\text{CO}}_{{3}}^{{{\mathbf{ \cdot }} - }}$$

13 $${\text{OH}} \cdot + {\text{ CO}}_{{3}}^{2 - } \to {\text{OH}}^{ - } + {\text{CO}}^{{{\mathbf{ \cdot }} - }}$$

To additionally scrutinize the CR degradation by the Co@N-PCNC/PMS system in practical applications, a real water matrix (tap water) was utilized. The tap water was obtained from the laboratory (catalysis laboratory, Refining Division, Egyptian Petroleum Research Institute). As shown in Fig. 7b, the removal of CR proceeds at a slower rate for the real water matrix compared to deionized water. Co@N-PCNC has a lower removal efficiency in real water matrices than in deionized water*,* which was presumably ascribed to the inhibitory effect exerted by the inorganic anions, which are inevitably present in real water matrices^89,90^.

#### Identification of reactive species

Aiming to reveal the nature of the reactive oxygen species radicals generated throughout the catalytic oxidation of CR, and hence the underlying mechanism, radical quenching investigations were conducted adopting methanol (MA) as a scavenger of SO_4_^**·**−^ and ·OH (Eqs. (14) and (15)) and tert-butyl alcohol (TBA) as an ^•^OH scavenger (Eq. (16)); the results are displayed in Fig. 6c. The results in Fig. 6c unveiled that the addition of TBA into the reaction mixture Co@N-PCNC/PMS system induced only a slight reduction in CR degradation efficiency; on the contrary, the degradation efficiency was substantially hindered and rapidly reduced from 98.4 to 36.4% upon the addition of MA. Nonetheless, a considerable CR degradation efficiency of more than 36.4% was still achieved after the use of MA and TBA. Consequently, a quenching experiment using l-histidine was also executed to reveal the role of singlet oxygen (^1^O_2_) in CR degradation (Fig. 7c). The CR degradation efficiency experienced a drastic decline to less than 5% upon adding l-histidine (0.1 M), insinuating that ^1^O_2_ played a major part in the degradation of CR in the Co@N-PCNC/PMS system.

14 $${\text{OH}} \cdot + \left( {{\text{CH}}_{3} } \right)_{3} {\text{COH }} \to {\text{CH}}_{2} \left( {{\text{CH}}_{3} } \right)_{2} {\text{COH}} \cdot + {\text{H}}_{2} {\text{O}}\quad \left( {{\text{K}} = \, 6.0 \, \times \, 10^{8} \;{\text{M}}^{ - 1} \;{\text{S}}^{ - 1} } \right)$$

15 $${\text{OH}} \cdot + {\text{CH}}_{3} {\text{OH}} \to {\text{CH}}_{2} {\text{OH}} + {\text{H}}_{2} {\text{O}}\quad \left( {{\text{K}} = \, 9.7 \, \times \, 10^{8} \;{\text{M}}^{ - 1} \;{\text{S}}^{ - 1} } \right)$$

16 $${\text{SO}}_{4}^{ - } + {\text{CH}}_{3} {\text{OH}} \to {\text{SO}}_{4}^{2 - } + {\text{CH}}_{2} {\text{OH}}\quad \left( {{\text{K}} = \, 1.0 \, \times \, 10^{7} \;{\text{M}}^{ - 1} \;{\text{S}}^{ - 1} } \right)$$

#### Kinetic study of CR degradation

The CR degradation kinetics using the Co@N-PCNC/PMS system were analyzed according to the pseudo-first-order (Eq. (17)) and Langmuir–Hinshelwood (L–H) (Eq. (18)) kinetic models.

17 $$\ln \, \left( {{\text{C}}_{{\text{t}}} /{\text{C}}_{{\text{o}}} } \right) \, = \, - {\text{K}}_{1} {\text{t}}$$

where K_1_ is the rate constant of the reaction (min^−1^), t is the time of the reaction (min), C_o_ and C_t_ are the concentrations of CR dye at time zero and t, respectively.

18 $$\frac{1}{{K_{1} }} = \frac{1}{{K_{c} K_{L - H} }} + \frac{{C_{o} }}{{K_{c} }}$$

where K_c_ is the surface reaction rate constant (mg L^−1^ min^−1^) and K_L–H_ is the adsorption equilibrium constant (L mg^−1^)^91^.

**Figure 8:**
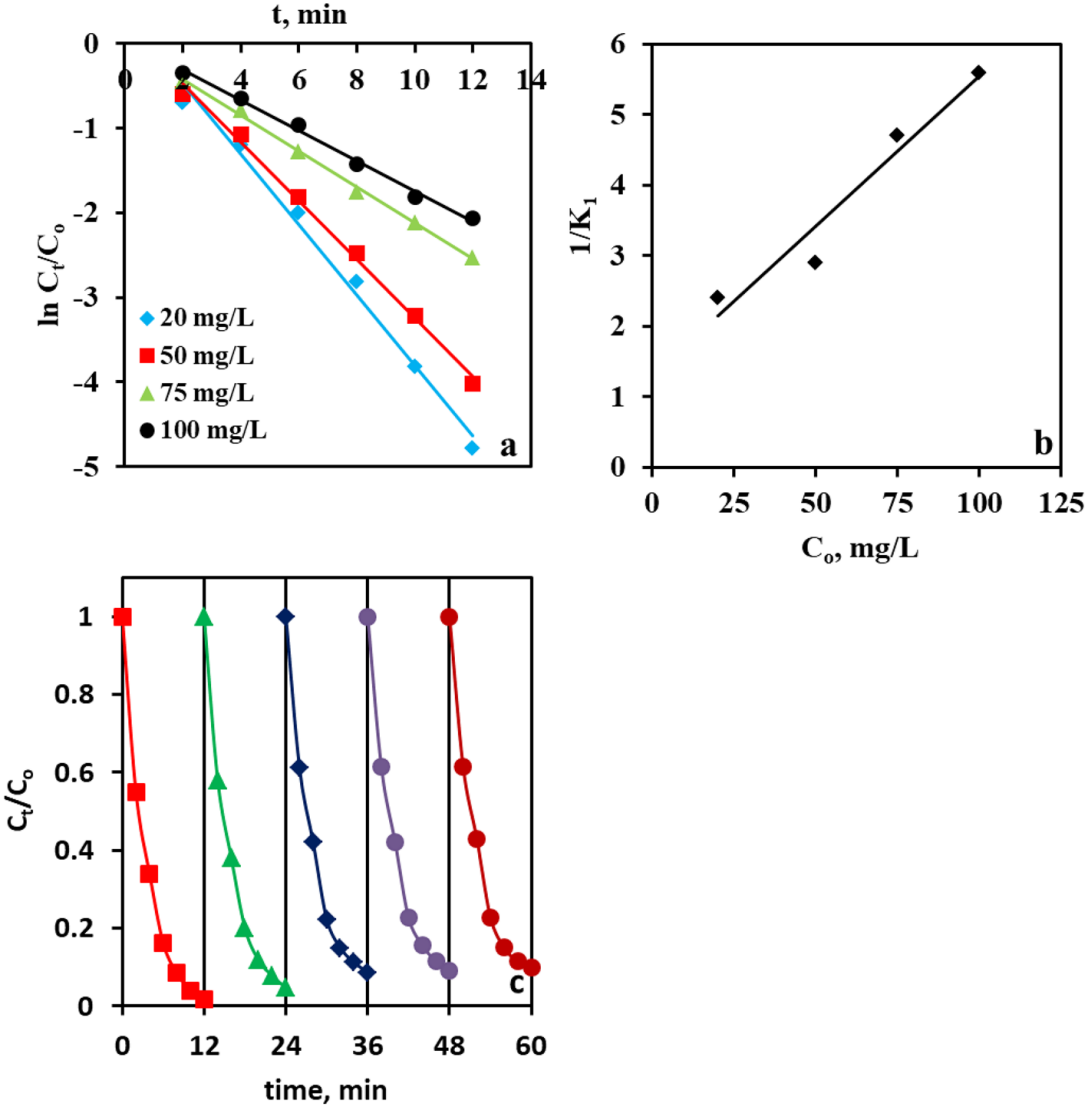
*Pseudo*-first-order plot (**a**), Langmuir–Hinshelwood plot (**b**), and reusability (**c**) of CR removal in Co@N-PCNC/PMS system.

Figure 8a depicts the plots of ln (C_t_/C_o_) vs. t at different CR initial concentrations from 25 to 100 ppm, which produce perfect straight lines with high R^2^ values (Table 2), ascertaining that the pseudo-first-order kinetic model is appropriate for the prediction of the kinetics of the CR degradation. The reaction rate constant (*K*_1_) values were computed from the slope of these plots and are compiled in Table 2. Please note that, as shown in Table 2, the increase in the initial CR concentration is associated with a drop in the values of the reaction rate constant. This drop is likely to arise from the restriction of the transportation of the PMS species from the reaction medium to the catalytically active centers, as well as the release of the produced active species from the solid boundary into the reaction medium by the augmented number of CR species^77^. In addition, the degradation of CR by Co@N-PCNC/PMS fits nicely into the L–H model, as inferred from the linear relationship between K^−1^ versus C_o_ (Fig. 8b) according to the L–H equation. From this plot, the values of K_c_ and K_L–H_ were determined as 23.5 mg L^−1^ min^−1^ and 0.0327 L mg^−1^, respectively.

**Table 2:** Kinetic parameters for the oxidative removal of CR with PMS using Co@N-PCNC.

C_o_, mg L^−1^	R^2^	K_1_
20	0.989	0.4154
50	0.995	0.3454
75	0.997	0.2123
100	0.994	0.179

#### Reusability

Stability and reusability can also be deemed crucial criteria for judging the practical feasibility of heterogeneous catalysts. This gives rise to their most conspicuous advantage over their homogeneous counterparts. Therefore, the recovery and reusability of our catalyst were scrutinized for five consecutive cycles in the PMS-assisted degradation of CR, and the results are depicted in Fig. 8c. The data in Fig. 8c demonstrated that the removal efficiency was progressively lowered after each recycling*.* The CR removal efficiency decreased from 98.45% in the 1st cycle down to approximately 90.21% in the 5th run, respectively. Please note that CR removal efficiencies of more than 90% were achieved after the fifth cycle*,* which verified the satisfactory stability and reusability of Co@N-PCNC. Notably, the used catalyst showed a similar diffraction pattern to that of the fresh one (Fig. S5). The lowering in CR degradation efficiency is likely to arise from the dissolution of Co from the surface of the catalyst during the oxidation process. Co-leaching analysis was also monitored after the 1st and 5th cycles. It was found that 0.56 and 0.14 ppm of Co were detected in the solution after the 1st and 5th cycles, respectively*.* These concentrations were lower than the maximum admissible content of Co in water resources of 1 ppm^9^. Moreover, the partial coverage of the active centers by CR and the reaction intermediates, as well as catalyst loss during the recovery and washing processes, can also account for the deteriorated efficiency upon repetitious usage^92^.

## Conclusions

In conclusion, the ZIF-67-derived carbon nanocubes encapsulated metallic Co NPs catalyst was successfully fabricated. The Co@N-PCNC catalyst exhibits superior activity for PMS activation to rapidly degrade CR dye. In addition, the Co@N-PCNC can still achieve satisfactory CR degradation efficiency of 78.6%, 82.9%, and 81.7% in 12 min, in the presence of Cl^−^, SO_4_^2−^, CO_3_^2−^, and NO_3_^−^ anions, respectively, thereby demonstrating that the Co@N-PCNC/PMS system can effectively resist the interference of the general inorganic anions. Eventually, the Co@N-PCNC showed good recyclability, and the CR removal rate was above 90% within 12 min after five cycles. These results demonstrated that the Co@N-PCNC has significant potential for practical application in refining dye-polluted effluents*.*

### Supplementary Information

Supplementary Information.

## Data Availability

All data generated or analyzed during this study are included in this published article.
