# Supplementary material for: Activation of peroxymonosulfate with ZIF-67-derived Co/N-doped porous carbon nanocubes for the degradation of Congo red dye
Source: Sci Rep. 2024 May 29;14:12313. doi: 10.1038/s41598-024-62029-8 (PMC11137160; doi:10.1038/s41598-024-62029-8)
Supplement: Supplementary file 1 — Supplementary Information. [file 41598_2024_62029_MOESM1_ESM.docx]

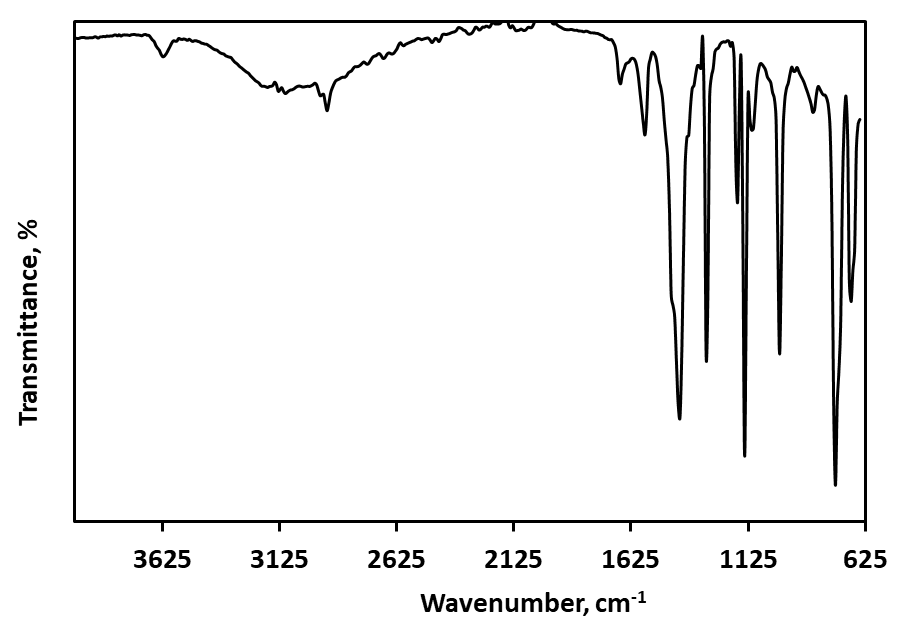


**Fig. S1.** FTIR spectra of ZIF-67.


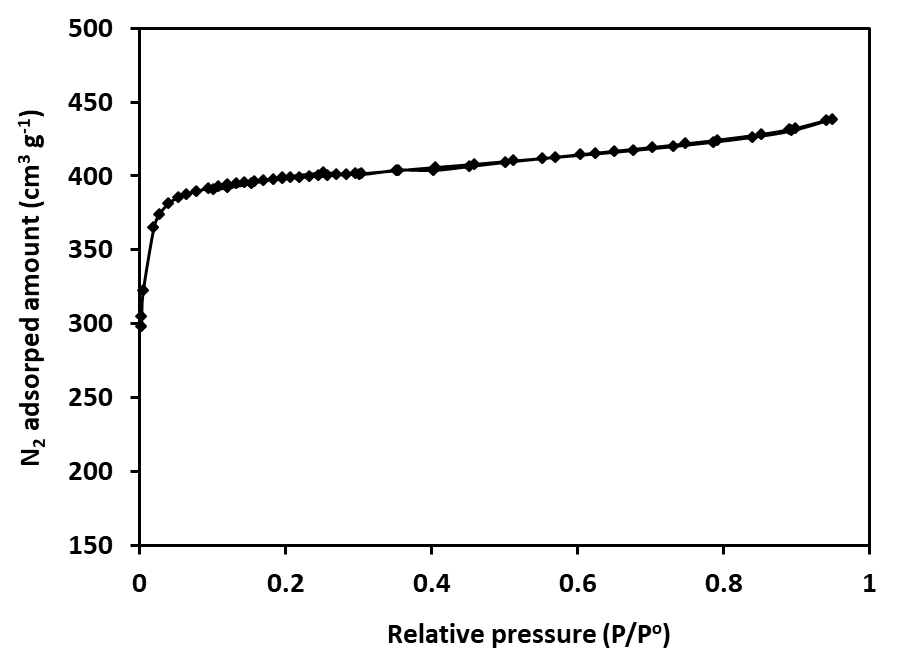


**Fig. S2.** N_2_ physisorption isotherm of ZIF-67.


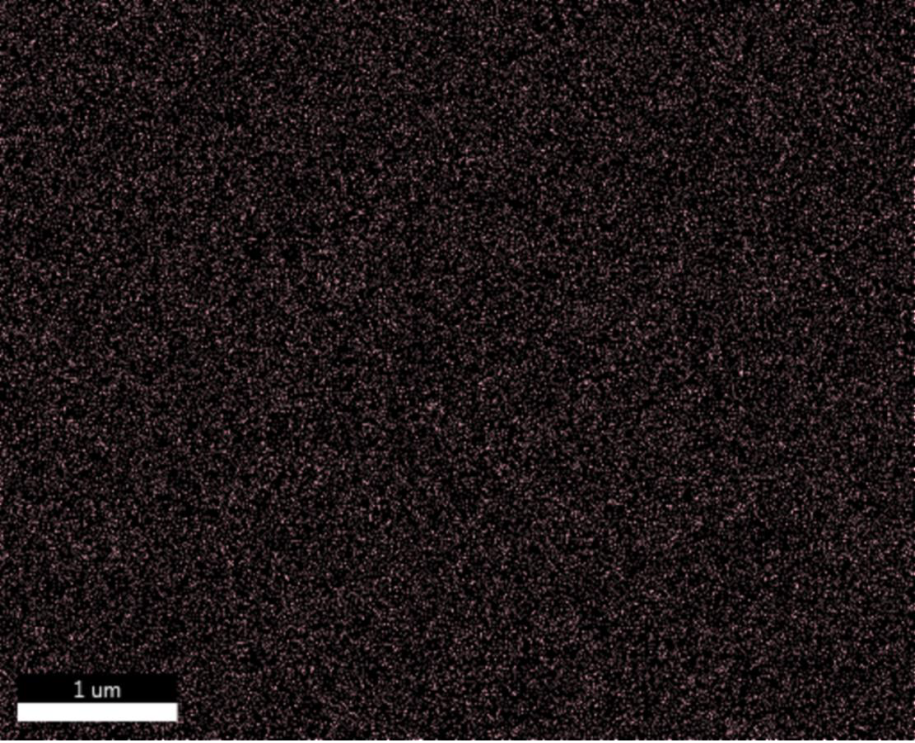


**(c)**

**Co**


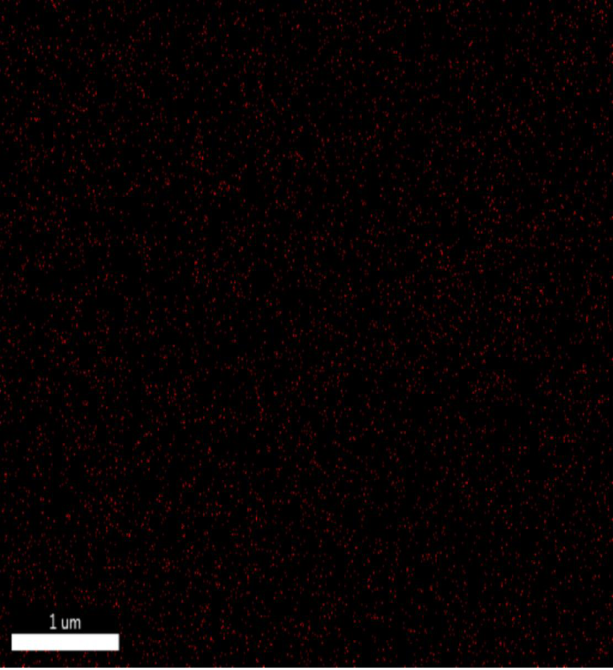


**(a)**

**C**


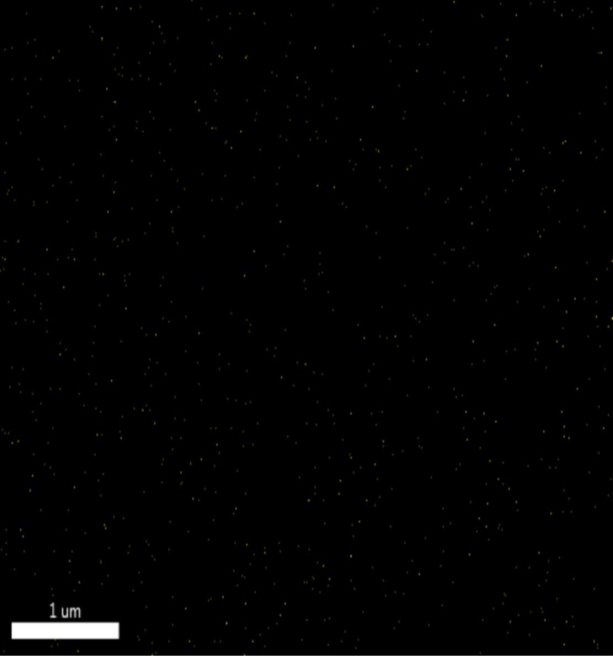


**(b)**

**N**


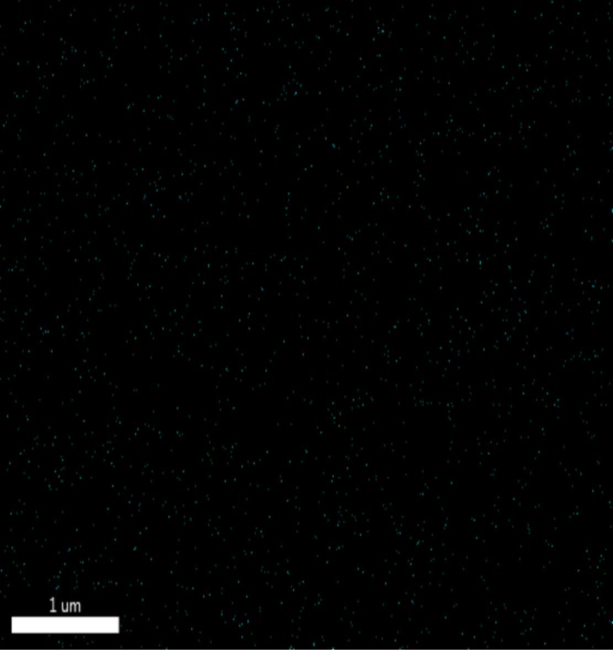


**(d)**

**O**

**Fig. S3.** EDS mapping and elemental analysis, including C (a), N (b), Co (c), O (d) elements.

# Table S1: Energy-dispersive X-ray spectroscopy (EDX) data of Co@N-PCNC.

| Element | Wt, % |
| --- | --- |
| C | 26.8 |
| N | 3.9 |
| O | 2.1 |
| Co | 67.2 |

**
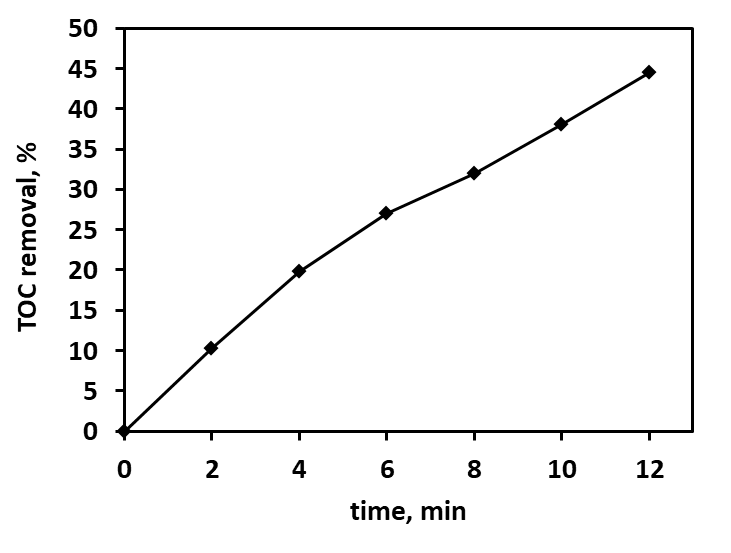
**

**Fig. S4.** TOC removal rate during the oxidation of CR at various time intervals.


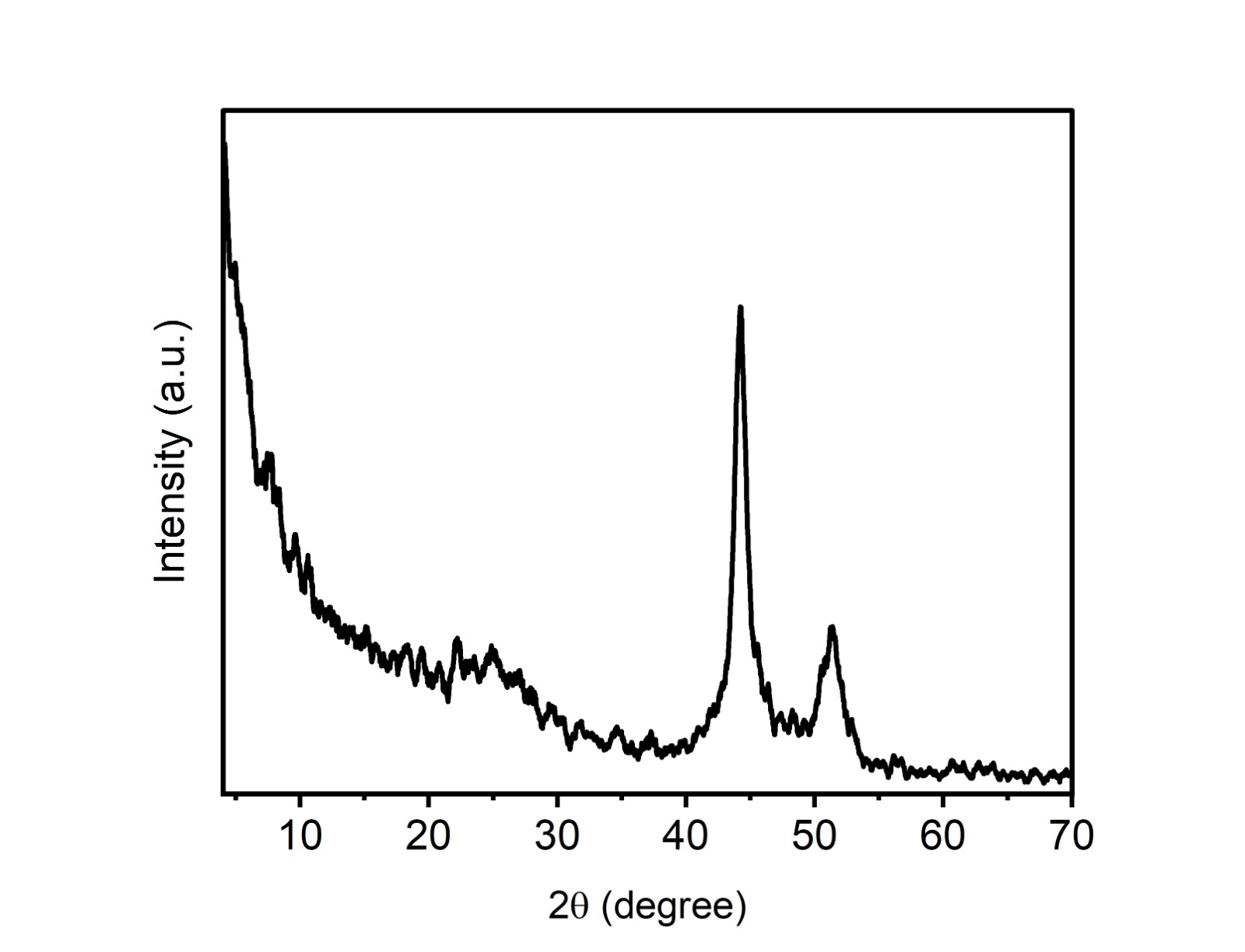


**Fig. S5.** XRD pattern of the used Co@N-PCNC.
